# Polyploid Giant Cancer Cells: A Distinctive Feature in the Transformation of Epithelial Cells by High-Risk Oncogenic HCMV Strains

**DOI:** 10.3390/v16081225

**Published:** 2024-07-31

**Authors:** Georges Herbein, Ranim El Baba

**Affiliations:** 1 Department Pathogens & Inflammation-EPILAB EA4266, University of Franche-Comté UFC, 25000 Besancon, France; ranim.elbaba@live.com; 2 Department of Virology, CHU Besançon, 250000 Besancon, France

**Keywords:** human cytomegalovirus, high-risk oncogenic strains, epithelial cells, oncogenesis, CTH cells, CTO cells, CTP cells, PGCCs, giant cell cycling

## Abstract

Human cytomegalovirus (HCMV) infection is common in tumor tissues across different types of cancer. While HCMV has not been recognized as a cancer-causing virus, numerous studies hint at its potential role in cancer development where its presence in various cancers corresponds with the hallmarks of cancer. Herein, we discuss and demonstrate that high-risk HCMV-DB and BL strains have the potential to trigger transformation in epithelial cells, including human mammary epithelial cells (HMECs), ovarian epithelial cells (OECs), and prostate epithelial cells (PECs), through the generation of polyploid giant cancer cells (PGCCs). A discussion is provided on how HCMV infection creates a cellular environment that promotes oncogenesis, supporting the continuous growth of CMV-transformed cells. The aforementioned transformed cells, named CTH, CTO, and CTP cells, underwent giant cell cycling with PGCC generation parallel to dedifferentiation, displaying stem-like characteristics and an epithelial–mesenchymal transition (EMT) phenotype. Furthermore, we propose that giant cell cycling through PGCCs, increased EZH2 expression, EMT, and the acquisition of malignant traits represent a deleterious response to the cellular stress induced by high-risk oncogenic HCMV strains, the latter being the origin of the transformation process in epithelial cells upon HCMV infection and leading to adenocarcinoma of poor prognosis.

## 1. Deciphering PGCCs in the Tumorigenic Landscape

Polyploid giant cancer cells (PGCCs) may possess either a single enlarged nucleus or multiple nuclei, denoted as mononucleated PGCCs or multinucleated PGCCs, respectively [1,2]. Initially, PGCCs were believed to arise solely from repeated failures in mitosis or cytokinesis, with limited proliferative potential; however, recent evidence has unveiled their resemblance to cancer stem cells (CSCs) [3]. PGCCs exhibit the ability to undergo asymmetric division, generating progeny cells expressing markers associated with epithelial–mesenchymal transition (EMT), thereby facilitating invasion and migration [4,5]. Various stimuli and stresses such as hypoxia, radiation, chemical drugs, viruses, and other stimuli that lead to DNA double-strand breaks can trigger PGCC formation [4].

PGCCs manifest across a spectrum of malignant solid tumors, encompassing melanoma, urothelial carcinoma, renal cell carcinoma, breast and ovarian cancer, pancreatic carcinoma, prostate carcinoma [4,6,7,8,9], as well as hematologic malignancies such as leukemia [10,11]. The abundance of PGCCs correlates with metastasis, resistance to chemoradiotherapy, and tumor recurrence [4]. Notably, PGCCs are frequently encountered in high-grade cancers. They play a pivotal role in driving tumor progression and influencing tumor diversity [12]. There is mounting evidence highlighting the presence of PGCCs in breast cancer (BC), ovarian cancer (OC), and prostate cancer (PCa), particularly in poor prognostic tumors where they function as stem-like cells capable of self-renewal and are considered prognostic indicators for tumor outcomes [4,5]. Due to their tumor-promoting functions, PGCCs are considered potential markers for predicting treatment response and patient outcomes in cancer and detecting their presence in biological fluids has become a new paradigm in cancer progression [5]. Efforts to develop treatments targeting PGCCs have shown promise in preclinical studies but have yet to translate into clinical practice [5,13].

Based on previous findings, herein, we aim to elucidate the role of human cytomegalovirus (HCMV) in infecting various epithelial cell types, including HMECs, OECs, and PECs, and generating PGCCs in long-term cultures. Further, we discuss the oncogenic potential of high-risk HCMV (HR-HCMV) strains (DB and BL) and their potential contribution to the development of triple-negative breast cancer (TNBC), OC, and PCa adenocarcinoma.

## 2. Unveiling the Viral Culprit: HCMV Orchestrating Cancer Pathogenesis

A growing body of research has shed light on the intricate relationship between HCMV infection and cancer development [14,15]. While traditionally viewed as a benign virus causing mild symptoms in healthy individuals, HCMV’s potential role as an oncogenic agent has garnered significant attention recently [6,8,16,17,18]. Numerous studies have implicated HCMV in the pathogenesis of various cancers, spanning a wide range of tissue types and malignancies. From glioblastoma to breast cancer, colorectal cancer, and prostate cancer, evidence continues to mount linking HCMV infection with tumor initiation, progression, and metastasis [19,20]. This association has prompted researchers to explore the intricate molecular mechanisms underlying HCMV-induced oncogenesis. In addition to its effect on individuals with immunosuppression and its direct transforming role, HCMV may promote tumor progression and metastasis, a concept referred to as oncomodulation [14]. For instance, HCMV infection enhanced tumor formation in mice implanted with neurospheres [21], it increased the malignant properties of the HepG2 liver cancer cell line [22], and EMT as well as WNT pathways were enhanced upon HCMV infection of colorectal cancer cells [23]. While oncomodulation might explain some of HCMV’s tumor-promoting effects, it does not fully account for the virus’s overall impact on the tumor and its surrounding microenvironment [14]. HCMV employs a multifaceted approach to drive oncogenesis, exploiting host cellular machinery to promote tumor growth and evade immune surveillance [14,24]. One mechanism involves the dysregulation of cell cycle control, leading to uncontrolled cellular proliferation, a hallmark of cancer [25]. Additionally, HCMV-encoded proteins interact with key signaling pathways involved in apoptosis inhibition, angiogenesis, and DNA repair, further fueling tumor progression [15]. Moreover, recent studies have highlighted the ability of HCMV to induce genomic instability and alter the tumor microenvironment, fostering a pro-tumorigenic milieu conducive to cancer growth and metastasis [26]. HCMV infection exhibits all of the recognized characteristics associated with cancer, such as continuous signaling for cell proliferation, the disruption of cellular metabolism, resistance to apoptosis, the promotion of genetic instability, the stimulation of blood vessel formation, the initiation of invasion and metastasis, fostering an environment of pro-tumor inflammation, facilitating indefinite cellular replication, evading immune-mediated destruction, unlocking phenotypic plasticity, nonmutational epigenetic reprogramming, the association with polymorphic microbiomes, and the appearance of senescent cells [14,27]. While various strains of HCMV may demonstrate some of these cancer-related traits, HR-HCMV strains are particularly adept at inducing invasion and metastasis, escaping growth inhibition and cellular contact controls, promoting sustained cellular dedifferentiation and stemness, fostering the formation of PGCCs, and driving oncogenic processes in tumors associated with poor prognoses [6,28,29].

Recent investigations have documented the prevalence of HCMV infection in tumor tissues across various malignancies, including malignant glioma, breast, colon, ovarian, liver, and cervical cancers, as well as prostate carcinoma [7,8,17,18,19,20]. Although HCMV has not yet been formally recognized as an oncogenic virus, numerous studies suggest its potential involvement in carcinogenesis as either an initiator or promoter, highlighting a symbiotic relationship between HCMV and tumors [14,28,30]. HCMV’s presence in various cancers aligns with the characteristics of cancer development outlined by Hanahan and Weinberg [27,31,32]. If HCMV gene products are indeed responsible for causing DNA damage, impairing DNA repair mechanisms, and stimulating proliferation in stem cells, then HCMV could potentially trigger the onset of cancer. While this notion has been suggested in the past, recent research has provided clearer evidence.

## 3. Epithelial Cells as a Target of HCMV Infection

While implicated in all aspects of cancer hallmarks as previously discussed, various HCMV proteins have the potential to initiate cellular transformation. Based on previous findings, the cellular transformation caused by HCMV was characterized by prolonged chronic cellular proliferation lasting from months to years, colony formation in soft agar, and the activation of pro-oncogenic pathways in infected cells, such as the Myc/EZH2 axis [6,7,8,9,16,17]. Early evidence has documented HCMV’s ability to induce the in vitro transformation of human embryonic lung fibroblasts and rodent fibroblasts [33,34]. Later on, the expression of HCMV immediate early (IE) proteins alongside the adenovirus E1A protein promoted cellular transformation through a “hit and run” mechanism, as IE expression was no longer detectable in the transformed cells [35]. In 2018, a study by our group was conducted showing the infection of human primary mammary epithelial cells (HMECs) with the clinical HCMV-DB strain which resulted in tumor colony formation in soft agar cultures. HCMV-DB transformed HMECs leading to tumor development in immunodeficient mice [16]. These findings validate the data from earlier studies, suggesting that only specific clinical strains of HCMV exhibit oncogenic properties and induce tumor development. A common characteristic of these viral strains appears to be their lack of rapid lytic infection, thereby allowing numerous virus-mediated mechanisms to act in an oncogenic manner without causing cell death, thus facilitating oncogenic transformation [14]. In primary HMECs infected with the clinical isolate HCMV-DB, the molecular prerequisites for oncogenic immortalization and transformation in vitro were met, namely p53 and Rb inactivation, telomere maintenance, the acquisition of constitutive mitogenic signals provided by Ras/cMyc, Akt activation, STAT3 activation, and cyclin D1 overexpression, resulting in enhanced cellular proliferation [6,16]. The transformed HMECs exhibited an HCMV-lncRNA4.9 signature and gave rise to rapidly growing triple-negative breast tumors when injected into NOD scid gamma (NSG) mice [16]. A similar HCMV RNA signature was detected in tumor biopsies of breast cancer patients compared to healthy breast tissue. All in all, our studies suggested a potential role of HCMV infection in the generation and development of a triple-negative breast cancer phenotype.

**Figure 1:**
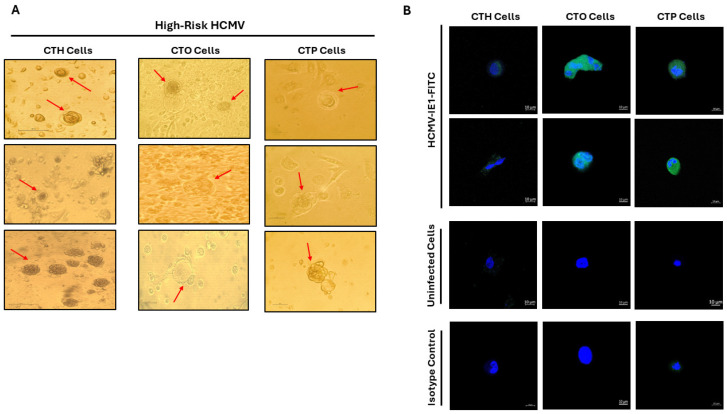
PGCC detection in CMV-transformed cells generated from HR-HCMV infection of HMECs, OECs, and PECs. (**A**). White light images of PGCCs generated in CTH, CTO, and CTP cultures. (**B**). Confocal microscopic images of IE1 and DAPI staining in CTH, CTO, and CTP cells. Magnification ×63; scale bar 10 μm. Red arrows represent the heterogeneous cellular morphologies detected in CTH, CTO, and CTP cell population.

**Figure 2:**
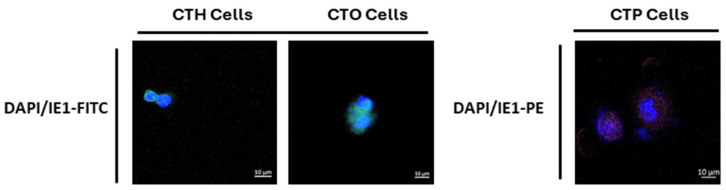
IE1 expression in CTH, CTO, and CTP cells. Confocal microscopic images of IE1 and DAPI staining in CTH, CTO, and CTP cells (two stained cells per image). Magnification ×63; scale bar 10 μm.

Additionally, to confirm the acute infection of HMECs, OECs, and PECs with HCMV-DB and BL, IE1 was detected in HMECs, ovarian epithelial cells (OECs), and prostate epithelial cells (PECs) infected with HCMV-DB and BL compared to uninfected epithelial cells, as previously shown by our research group [6,7,8,9]. We also observed the presence of enlarged cells with large nuclei exclusively in HMECs, OECs, and PECs under chronic infection by high-risk HCMV-DB and BL strains contrasting with uninfected epithelial cells [6,8,9]. The morphological heterogeneity included blastomeres and blastocytes, multinucleated cells, mesenchymal cells, cellular connections between HMECs, OECs, and PECs, as well as atypical morphologies. These emerging cells were designated as “CMV-transformed mammary epithelial cells” or CTH cells, “CMV-transformed ovarian epithelial cells” or CTO cells, and “CMV-transformed prostate epithelial cells” or CTP cells [6,8,9,16] (Figure 1 and Figure 2). Notably, colony formation was observed in soft agar cultures containing CTH, CTO, and CTP-DB and BL, in contrast to uninfected HMECs, OECs, and PECs. At the proteomic level, increased expression of EZH2 and Myc was identified in CTH, CTO, and CTP-DB and BL, in comparison to uninfected cells. After evaluating the proliferative capacity of HMECs, OECs, and PECs persistently infected with high-risk HCMV strains, an increase in Ki67Ag expression was observed in CTH, CTO, and CTP-DB and BL cells, especially in PGCCs [6,7,8,9]. Collectively, these findings imply that certain HCMV strains, namely HR-HCMV, may possess not only oncomodulatory capabilities but, more importantly, genuine oncogenic and tumor-promoting mechanisms in specific cellular contexts.

## 4. Linking HCMV Infection to PGCC Formation in Epithelial Cells

The recent data produced by our research group indicated that high-risk HCMV strains transform several epithelial cell types, along with generating PGCCs in vitro [6,28,36]. Evidence from in vitro and in vivo data indicated that HCMV might play a role in the onset or progression of breast cancer [6,16]. Further, studies demonstrated the presence of HCMV proteins and DNA in breast cancer and sentinel lymph node metastatic tissues [37]. In previous studies, we observed that infection with the clinical high-risk strains HCMV-DB and HCMV-BL led to the transformation of HMECs into CTH cells [6,16,29]. These transformed cells exhibited characteristics such as a polyploid phenotype, stemness, and EMT plasticity, ultimately resulting in the formation of rapidly growing breast tumors in NSG mice [16]. Moreover, a potential association was discovered between HCMV, PGCCs, and EZH2, as well as their potential interaction with Myc in the context of breast cancer development [7,17]. Additionally, morphological and phenotypic features of CTH cells undergoing the giant cell cycle were detected upon HCMV infection, in addition to the potential involvement of Myc and EZH2 in both CTH cells and breast cancer biopsies [6,7,17,29].

In the context of OC, which is the most prevalent type of cancer among gynecologic malignancies, studies revealed that HCMV proteins and nucleic acids were frequently detected at different levels in high-grade serous ovarian cancer (HGSOC) [38]. Previously, a study reported HCMV’s role as a reprogramming agent, leading to the transformation of OECs into “CMV-transformed Ovarian cells” (CTO). HCMV infection of OECs with these high-risk HCMV strains created a pro-oncogenic environment, leading to sustained growth of CMV-transformed OECs and the formation of colonies in soft agar. The CTO cells exhibited dedifferentiation, stemness, and a hybrid EMT-MET phenotype, ultimately resulting in the generation of PGCCs and the formation of spheroids. The presence of HCMV, coupled with polyploidy, EZH2 upregulation, and the acquisition of a malignant phenotype, supports the transformation process. In vivo, 72% of HGSOC biopsies harbored HCMV, with elevated counts of PGCCs and enhanced EZH2 expression, indicating a strong correlation between HCMV, PGCCs, and EZH2 expression. Further, three HCMV strains isolated from EZH2-high HGSOC tumors induced the transformation of OECs into CTO cells, characterized by increased expression of EZH2, Ki67Ag, and Myc, along with the induction of polyploidy [8].

Additionally, previous findings indicated that HCMV seropositivity is associated with an increased risk of prostate cancer, which is the most prevalent malignancy in men, with high mortality rates, especially in advanced stages [39,40]. There is increasing interest in exploring viral factors in prostate cancer (PCa) development, particularly focusing on HCMV [9]. In addition, PGCCs were observed in PCa tissues and play a role in tumor diversity, recurrence, and resistance to treatment [41,42]. Research suggested that HCMV may directly contribute to PCa development by inducing PGCC formation and triggering cancer stemness and EMT processes [9]. Thus, understanding the relationship between HCMV infection and PCa pathogenesis could offer insights into potential therapeutic targets and diagnostic approaches. A previous study showed that the heightened expression of Myc and EZH2 induced by HCMV, coupled with the emergence of stemness and EMT cellular traits in PECs expressing IE1, resulted in the formation of CMV-transformed prostate cells (CTP cells) [9]. This observation suggests a potentially important model for understanding prostate cancer. Additionally, patients with prostate cancer exhibiting focal pleomorphic giant cells experienced decreased overall survival, underscoring the significant involvement of PGCCs in cancer advancement [43,44]. Further investigations are warranted to ascertain whether PGCCs identified in urine could serve as an early cancer marker and whether they hold a prognostic value [45].

**Figure 3:**
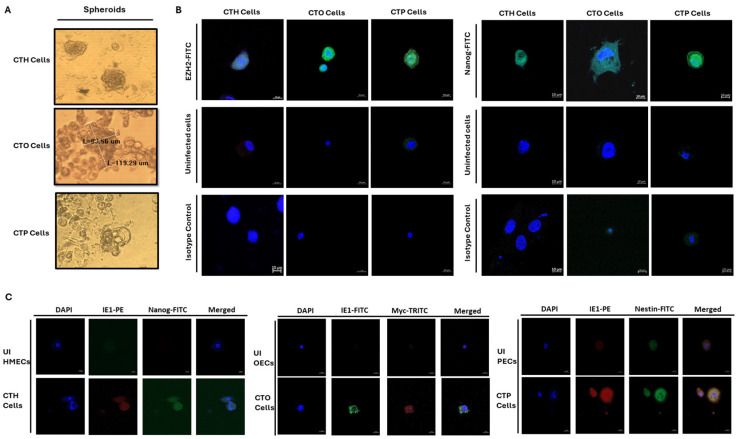
Stemness traits in PGCCs elicited from HR-HCMV infection of HMECs, OECs, and PECs. (**A**) Spheroids in CTH, CTO, and CTP cultures. As specified, the size of a CTO spheroid is approximately 100 μm. (**B**) EZH2 and Nanog expression by confocal microscopy in CTH, CTO, and CTP cells. Magnification ×63; scale bar 10 μm. (**C**). Confocal microscopy images showing IE1/Nanog co-staining for CTH cells, IE1/Myc co-staining for CTO cells, and IE1/Nestin co-staining for CTP cells. Uninfected HMECs, OECs, and PECs were used as controls; magnification ×63 and scale bar 10 μm.

In our research, we observed the generation of PGCCs by CMV-transformed cells, including CTH, CTO, and CTP cells, which displayed a heterogeneous population exhibiting distinct morphological characteristics such as budding, filopodia, cells filled with lipid droplets, blastomere-like cells, and multinucleated cells. These features are commonly observed in breast cancer, ovarian cancer, and prostate cancer. CMV-transformed cells bearing PGCCs acquired characteristics resembling embryonic stem cells (Figure 3) and exhibited a phenotype combining traits of EMT and stemness (Table 1) [6,7,8,9]. Studies have demonstrated that the acquisition of EMT and stemness properties enhances the invasiveness and metastatic potential of cancer cells within tumors [46,47]. Compared to uninfected cells, CMV-transformed cells infected with the DB and BL strains showed increased expression of EZH2, Myc, Ki67Ag, SOX2, Nanog, CD44, vimentin, Nestin, and CD49f, promoting the abovementioned phenotypes (Table 1) [6,7,8,9]. As reported previously, the aforementioned stemness and EMT markers were linked to the presence of viral proteins (for instance HCMV-IE1) in CMV-infected cells, such as CTO and CTP cells [8,9]. Thus, mainly IE1-expressing cells displayed EMT and stemness properties compared to the uninfected cells in the culture. IE1/Nanog, IE1/Myc, and IE1/Nestin were concomitantly expressed in CTH, CTO, and CTP cells, respectively (Figure 3C). It is also of high importance to analyze the whole cell population of epithelial cells (HMECs, OECs, and PECs) infected with high-risk oncogenic HCMV strains for dual and/or multiple staining, showing the expression of IE1 and the oncogenic, stemness as well as EMT markers by flow cytometry, as previously shown for human astrocytes [18], leading to the generation of CMV-transformed cells in the culture [6,8,9,18]. The presence of HCMV, coupled with the occurrence of polyploidy, the upregulation of EZH2, and the acquisition of malignant features, strongly suggests a transformation process [6,8,9]. All in all, these findings suggest that polyploidy induced by HCMV may contribute to the acquisition of a malignant phenotype through the cycling of giant cells, especially in adenocarcinoma.

Previously, we have isolated two HCMV strains from TNBC biopsies and three HCMV strains from HGSOC biopsies [7,8]. These clinical HCMV strains displayed oncogenic and stemness features when cultivated on HMECs and OECs. The transformed HMECs and OECs generated CTH and CTO cells, respectively, which promoted PGCC formation in vitro [7,8,17,48]. Altogether, the five high-risk HCMV strains that were isolated from tumor biopsies played a role in triggering polyploidy, thereby leading to tumor aggressiveness.

Generally, HCMV infection, resembling oncoviruses, could be implicated in the pathogenesis of adenocarcinoma [36,49]. HCMV can establish persistent infections in epithelial cells, leading to chronic inflammation and tissue damage, both of which are known contributors to carcinogenesis [50]. In previous studies, HCMV-IE1 was detected in long-term cultures of CTH, CTO, and CTP cells, confirming the sustained HCMV replication. The virus possesses oncogenic and stemness properties that dysregulate the cellular signaling pathways involved in cell growth, proliferation, and apoptosis, facilitating the transformation of normal epithelial cells into cancerous cells. CMV-transformed cells, including CTH, CTO, and CTP cells, retained stemness and EMT phenotypes, reinforcing the link between HCMV and oncogenesis [6,7,8,9]. High-risk HCMV strains trigger substantial stress within infected cells which, in turn, acts as a catalyst for transformation. Further, in epithelial cells, a hallmark of HCMV-induced transformation is the occurrence of giant cell cycling. Herein, the formation of PGCCs was a critical indicator of cellular transformation and was closely associated with the pathogenic effects of HCMV. Additionally, the Myc/EZH2 pathway represented a significant molecular mechanism through which high-risk HCMV strains exerted their transforming effects. Collectively, these interactions between HCMV infection, epithelial cells, and adenocarcinoma underscore the complex interplay between viral and host factors in the development and progression of cancer, especially breast cancer, ovarian cancer, and prostate cancer. The critical role of PGCCs in the generation and development of numerous adenocarcinomas far beyond breast, ovarian, and prostate adenocarcinoma and PGCCs’ position at the crossroads of oncogenesis and infectious agents including HCMV have just started to emerge.

## 5. Implications and Future Directions

Identifying the role of HCMV in PGCC formation may open up new avenues for therapeutic intervention. Targeting HCMV or the pathways involved in PGCC formation could potentially lead to the development of novel cancer treatments [5,13,15]. Most importantly, understanding PGCC formation pathways has led to the development of therapies with preclinical efficacy [4]. Thus, targeting key proteins or signaling pathways as well as identifying immune and metabolic biomarkers involved in PGCC formation presents novel therapeutic avenues for combating tumors [1,5,13]. PGCCs exhibit a higher metabolic rate, making them susceptible to glycolysis inhibitors and drugs targeting the mTOR pathway. Additionally, sphingolipid metabolism alterations in PGCCs present further therapeutic opportunities. Pharmacological approaches targeting key players in the endoreplication cycle are also being explored [5]. Recently, single-cell morphological and transcriptome analysis has revealed promising anti-PGCC strategies for breast cancer treatment and other malignancies [13]. All in all, anti-PGCC therapeutic development could enhance clinical outcomes, especially given the correlation between PGCC abundance and high tumor grades. On the other hand, while progress has been made in the development of anti-HCMV drugs (such as ganciclovir, letermovir, cidofovir, maribavir, foscarnet, etc.) and vaccines (for instance, DNA, mRNA, plasmid-based, virus-vectored vaccines, etc.), further research is needed to improve their efficacy, safety, and accessibility, particularly for populations at high risk of HCMV-associated cancers [15,51,52]. As well, it is worth mentioning the impact of immunotherapy utilizing vaccination and ex vivo T-cell expansion to boost the immune reaction against CMV-associated tumors [50,53]. Despite promising results, further research and efforts are needed to address potential challenges.

## 6. Conclusions

We conclude that high-risk oncogenic HCMV strains, with elevated EZH2/Myc expression, have the ability to generate PGCCs, the dedifferentiation of epithelial cells, as well as the emergence of stem-like features and EMT/MET traits alongside giant cell cycling. Further investigations with comprehensive analyses could provide insights into the intricate development of breast, ovarian, and prostate adenocarcinoma, potentially exploring innovative targeted treatment approaches without neglecting the central role played by PGCCs that are generated by high-risk oncogenic HCMV strains.

## Figures and Tables

**Table 1:** Biological characteristics of PGCCs detected in CTH, CTO, and CTP cultures.

	CTH Cells	CTO Cells	CTP Cells
**PGCCs’** **Characteristics**	Single- and multinucleated giant cells with small budding cells [6,7,17].	Single- and multinucleated giant cells with small budding cells. Lipid droplet-filled giant cells [8].	Single- and multinucleated giant cells with budding cells (usually with protrusions). Lipid droplet-filled giant cells [9].
**HCMV gene** **detection**	IE1, UL69, and RNA4.9 [6,7,17]	IE1, UL69 [8]	IE1 [9]
**HCMV protein** **detection**	IE1,pp65, and late antigen [6,7,17]	IE1 [8]	IE1 [9]
**Polyploidy analysis by FACS**	Tetraploidization and polyploidy (≥4 N) [6,7,17]	Tetraploidization and polyploidy (≥4 N) [8]	Polyploidy (>4 N) [9]
**Stemness phenotype**	EZH2, Suz12, Nanog, Oct4, Sox2, SSEA4 [6,7,17]	EZH2, Sox2, Nanog, Suz12, Oct4 [8]	EZH2, Nestin, Nanog, Sox2 [9]
**EMT markers**	Vimentin, CD44, CD49f [6,7,17]	Vimentin, CD44, CD49f [8]	Vimentin [9]

## Data Availability

Not applicable.

## Abbreviations

BC	Breast cancer
CSCs	Cancer stem cells
CTH cells	CMV-transformed mammary epithelial cells
CTO cells	CMV-transformed ovarian epithelial cells
CTP cells	CMV-transformed prostate epithelial cells
EMT	Epithelial–mesenchymal transition
HGSOC	High-grade serous ovarian cancer
HR-HCMV	High-risk HCMV
HCMV	Human cytomegalovirus
HMECs	Human primary mammary epithelial cells
IE	Immediate early
NSG	NOD scid gamma
OC	Ovarian cancer
OECs	Ovarian epithelial cells
PGCCs	Polyploid giant cancer cells
PCa	Prostate cancer
PECs	Prostate epithelial cells
TNBC	Triple-negative breast cancer
